# Genome Sequence of the Lanthanide-Responsive Methylotrophic Bacterium Methylorubrum extorquens Strain GM97

**DOI:** 10.1128/mra.00268-23

**Published:** 2023-06-29

**Authors:** Kohei Nakamura, Kosuke Mizuno, Masaya Shimada, Takashi Hayakawa, Tomoyuki Nakagawa

**Affiliations:** a Faculty of Applied Biological Sciences, Gifu University, Gifu, Japan; b The Graduate School of Natural Sciences and Technologies, Gifu University, Gifu, Japan; c Department of Applied Life Studies, College of Nagoya Women’s University, Aichi, Japan; The University of Arizona

## Abstract

We present the complete genome sequence of Methylorubrum extorquens strain GM97, which formed large colonies on a 1/100 nutrient plate with samarium (Sm^3+^). The genome for strain GM97 was estimated to be 7,608,996 bp, which suggests that the strain is closely related to *Methylorubrum extorquens* strains.

## ANNOUNCEMENT

Strain GM97, which shows lanthanide (Ln)-dependent methylotrophic growth, was isolated from a weed leaf in Gifu, Japan, by enrichment culture using methanol/La^3+^ Hypho medium (1). A single colony of strain GM97 was picked on the methanol/La^3+^ Hypho plate, and the strain was identified as Methylorubrum extorquens using the 16S rRNA gene sequence. Strain GM97 formed large colonies on a 1/100 nutrient plate with samarium (Sm^3+^) (2). In this study, we report the M. extorquens strain GM97 genome sequence.

Genomic DNA from M. extorquens strain GM97 grown on methanol/La^3+^ Hypho medium was isolated using the NucleoSpin soil kit (Macherey-Nagel GmbH & Co. KG, Düren, Germany). Library preparation and sequencing were conducted by Macrogen (Seoul, South Korea) using the PacBio RS II platform with a 20-kb insert size. Genomic DNA (8 μg) was sheared using the g-TUBE device (Covaris Inc., Woburn, MA, USA) and purified using AMPure PB magnetic beads. The library and SMRTbell templates were prepared using the PacBio DNA template prep kit 1.0 and the PacBio DNA/polymerase binding kit P6, respectively. The PacBio DNA sequencing kit 4.0 and single-molecule real-time (SMRT) cell 8Pac were used for sequencing. Sequencing generated 285,195 reads (1.12 Gb), with a read length *N*_50_ value of 5,447 bp.

Default parameters were used for all software unless otherwise specified. HGAP v3.0 (3) was used to filter the reads, perform *de novo* assembly, and polish the assembly using default parameters, generating a closed complete genome sequence 5,472,957 bp long with a G+C content of 68.4%. Annotation of the genome and prediction of the replication origin were performed using the DFAST pipeline (4). Prodigal implemented within the DFAST pipeline was selected for coding DNA sequence (CDS) annotation. The genome contains 5,016 CDSs, 15 rRNAs, 63 tRNAs, and no CRISPR arrays. Average nucleotide identity (ANI) analysis (5) indicated that M. extorquens AM1 (GenBank accession number GCF_000022685.1) is closest to strain GM97, showing an ANI value of 99.41%. The following two sets of methanol dehydrogenase clusters were found in the genome: (i) the *xox* cluster, encoding Ln-dependent methanol dehydrogenase (MSPGM_18470-MSPGM_18490), and (ii) the *mxa* cluster, encoding Ca-dependent methanol dehydrogenase and its related accessory proteins (MSPGM_41370-MSPGM_41500), similar to those found in M. extorquens AM1 (6). Both clusters encoded all proteins, showing 100% homology to proteins encoded by the *xox* and *mxa* clusters on the strain AM1 genome when subjected to a search using blastp.

The available information for the strain GM97 genome will help to elucidate new mechanisms for Ln-dependent phenotypes of the genus *Methylorubrum*.

### Data availability.

The complete genome sequence of *Methylorubrum extorquens* GM97 has been deposited in the DDBJ under the accession number AP025942.1. The version described in this paper is version AP025942.1. The raw sequence reads were deposited in the DDBJ Sequence Read Archive under the accession number DRA014087, the BioSample accession number SAMD00469826, and the BioProject accession number PRJDB13504.
